# O‐GlcNAcylation promotes colorectal cancer progression by regulating protein stability and potential catcinogenic function of DDX5

**DOI:** 10.1111/jcmm.14038

**Published:** 2018-11-28

**Authors:** Nan Wu, Mingzuo Jiang, Yuying Han, Haiming Liu, Yi Chu, Hao Liu, Jiayi Cao, Qiuqiu Hou, Yu Zhao, Bing Xu, Xin Xie

**Affiliations:** ^1^ Laboratory of Tissue Engineering, Faculty of Life Science Northwest University Xi’an Shaanxi China; ^2^ State Key Laboratory of Cancer Biology National Clinical Research Center for Digestive Diseases and Xijing Hospital of Digestive Diseases, Air Force Medical University (Fourth Military Medical University) Xi’an Shaanxi China; ^3^ College of Computer Science and Technology Jilin University Changchun Jilin China; ^4^ Department of Gastroenterology Second Affiliated Hospital of Xi'an Jiao tong University Xi’an Shaanxi China

**Keywords:** AKT, Colorectal cancer, DDX5, mTOR, O‐GlcNAcylation

## Abstract

The RNA helicase p68 (DDX5), a key player in RNA metabolism, belongs to the DEAD box family and is involved in the development of colorectal cancer. Here, we found both DDX5 and O‐GlcNAcylation are up‐regulated in colorectal cancer. In addition, DDX5 protein level is significantly positively correlated with the expression of O‐GlcNAcylation. Although it was known DDX5 protein could be regulated by post‐translational modification (PTM), how O‐GlcNAcylation modification regulated of DDX5 remains unclear. Here we show that DDX5 interacts directly with OGT in the SW480 cell line, which is the only known enzyme that catalyses O‐GlcNAcylation in humans. Meanwhile, O‐GlcNAcylation could promote DDX5 protein stability. The OGT‐DDX5 axis affects colorectal cancer progression mainly by regulating activation of the AKT/mTOR signalling pathway. Taken together, these results indicated that OGT‐mediated O‐GlcNAcylation stabilizes DDX5, promoting activation of the AKT/mTOR signalling pathway, thus accelerating colorectal cancer progression. This study not only reveals the novel functional of O‐GlcNAcylation in regulating DDX5, but also reveals the carcinogenic effect of the OGT‐DDX5 axis in colorectal cancer.

## 1 INTRODUCTION

The RNA helicase p68 (DDX5) belongs to the DEAD box family and plays a key role in RNA metabolism. Growing evidences revealed that DDX5 is significantly associated with tumorigenesis.^1^, ^2^ It is overexpressed in colorectal^3^, gastric^4^, breast, and prostate tumours and is associated with tumour progression.^5^, ^6^ In addition, it acts as a coactivator of several transcription factors, including β‐catenin, p53, oestrogen receptor alpha, AKT signalling pathway, c‐Myc, and androgen receptors.^7^, ^8^ Recent study also showed that DDX5 is significantly elevated in gastric cancer and co‐excited the mTOR signalling pathway to enhance the growth of gastric cancer cells.

Protein O‐GlcNAcylation is a broad and dynamic modification of β‐N‐acetyl‐D‐glucosamine.^9^, ^10^, ^11^, ^12^ It is a specific type of post‐translational modification catalysed by O‐linked N‐acetylglucosamine transferase (OGT), resulting in the transfer of O‐linked β‐N‐acetylglucosamine (O‐GlcNAcylation) to the serine of the target protein or threonine residue.^9^, ^13^ Previous studies have shown that it can induce conformational changes to initiate protein folding, compete with phosphorylation of the same or proximal serine or threonine, and disrupt protein‐protein interactions. And regulate protein stability.^14^, ^15^, ^16^, ^17^ Pathologically, abnormal O‐GlcNAcylation has been shown to stimulate tumorigenesis in various cancers by modulating cell signalling, transcription, cell division, metabolism, and cytoskeletal regulation.^12^, ^18^, ^19^, ^20^

Colorectal cancer (CRC) is one of the major health problems with high mortality worldwide.^21^ Epidemiological studies have shown a strong association between O‐GlcNAcylation and poor prognosis of CRC.^22^ In CRC patients, the synthesis of UDP‐N‐acetyl‐D‐glucosamine (UDP‐GlcNAc) is increased, which is the substrate for OGT for O‐GlcNAcylation of target proteins and O‐GlcNAcylation of certain proteins may be a key factor in tumorigenesis.^23^ In addition, abnormal activation of AKT/mTOR signalling is the key factor of CRC, which leads to tumour growth and metastasis.^24^, ^25^, ^26^ Previous studies have shown that DDX5 can transcriptionally co‐activate AKT signaling pathway in CRC to regulate cancer progression.^7^ Because of the significant increase in the prevalence of CRC, it is important to understand the molecular basis underlying this disease and the potential role of DDX5 and O‐GlcNAcylation in CRC.

In this study, we demonstrated that O‐GlcNAcylation of DDX5 is involved in colorectal cancer progression by activation of AKT/mTOR signalling pathway, which opens up a new sight for the treatment of colorectal cancer.

## 2 MATERIALS AND METHODS

### 2.1 Cell lines and cell culture

Human CRC cell lines HT29, HCT116, SW480, SW620, and normal intestinal epithelial cells NCM460 were obtained from the Chinese Academy of Sciences Cell Bank (China). NCM460, HT29, HCT116, SW480, and SW620 cells were cultured in DMEM medium supplemented with 10% (v/v) FBS (Gibco) and 1% penicillin/streptomycin. All cells were cultured at 37°C with 5% (v/v) CO_2_. All cells were tested for mycoplasma contamination.

### 2.2 RNA extraction, reverse transcription, and real‐time RT‐PCR

Total RNA from cells was extracted using an RNA isolation kit (Qiagen, Germany) according to the manufacturer's instructions. Subsequently, the RevertAid First Strand cDNA Synthesis Kit (TaKaRa, Japan) was used to reverse‐transcribe the messenger RNA (mRNA) from the total mRNA; the specific primer (Table S1) and the SYBR premix Ex Taq (TaKaRa) were used to expand by real‐time qPCR. It was carried out with the following parameters: pre‐denaturation at 95°C for 5 minutes, denaturation at 95°C for 10 seconds, annealing at 62°C for 20 seconds, and extension at 72°C for 30 seconds for 40 cycles. GAPDH was used as an internal control.

### 2.3 Cycloheximide or Thiamet‐G treatment

Cycloheximide (Sigma‐Aldrich, USA) was dissolved in dimethyl sulfoxide (Sigma‐Aldrich) to a concentration of 500 mg/mL and diluted in DMEM to a final concentration of 50 mg/mL. Thiamet‐G (Calbiochem, San Diego, USA) was dissolved in DMSO to a concentration of 40 mmol/L and diluted to a final concentration of 10 μmol/L in DMEM medium. Cells were incubated with DMEM medium containing 10% FBS and 1% penicillin/streptomycin to achieve a cell concentration of 80% prior to cycloheximide or Thiamet‐G treatment. Next, cells were treated with cycloheximide or Thiamet‐G (10 μmol/L) in the absence of FBS for 12 hours.

### 2.4 Western blotting

For Western blot (WB), the protein was resolved on SDS‐polyacrylamide gel electrophoresis (SDS‐PAGE) gel followed by standard WB. The primary antibodies used were anti‐GAPDH (CST, #5174, 1:2000), anti‐β‐Actin (CST, #3700, 1:2000), anti‐OGT (Abcam, #ab184198, 1:1000), anti‐Flag tag (Sigam,#F2555), 1:1000), anti‐DDX5 (Santa Cruz, #sc‐166167, 1:1000), anti‐O‐GlcNAcylation (Abcam, #ab2739, 1:1000), anti‐AKT (CST, #4691, 1:1000), anti‐p‐AKT (CST, #4060, 1:1000), anti‐mTOR (CST, #2972, 1:1000), and anti‐p‐mTOR (CST, #5536, 1:1000). Observe proteins with enhanced chemiluminescence reagents.

### 2.5 Co‐immunoprecipitation

The cells were collected and lysed with IP lysis buffer (Thermo Scientific, #87787) for 1 hour. Cell lysates were collected and incubated overnight with PureProteome Protein A or Protein G Magnetic Beads (Millipore, #LSKMAGA02) and antibodies at 4°C; immunoprecipitates were washed five times and then subjected to immunoblot analysis. Antibodies for IP are anti‐OGT (Abcam, #ab184198, 1:100), anti‐DDX5 (Santa Cruz, #sc‐166167, 1:100), and anti‐O‐GlcNAcylation (Abcam, #ab2739, 1:100).

### 2.6 Cell viability assay

The cells were seeded in a 96‐well plate (1 × 10^4^ cells/well), and cultured in a 37°C, 5% CO_2_ humidified incubator for 24 hours. Ten microlitres of Cell Counting Kit‐8 solution (Dojindo, Kumanoto, Japan) was added to each well and incubated for 2 hours at 37℃ in a 5% CO_2_ humidified incubator. Spectrometer Varioskan^®^ Flash (Thermo Fisher, Waltham, USA) was used to measure absorbance at 450 nm. A proliferation curve is drawn in which time is taken as the abscissa and the average absorbance value in each group is taken as the ordinate. The experiment was performed in triplicate.

### 2.7 Colony formation assay

The cells were seeded in 6‐well plates (500 cells/well) and incubated at 37℃ in a 5% CO_2_ humidified incubator. After 2 weeks, the cells were stained with Gentian violet (Beyotime Biotechnology, Shanghai, China). The experiment was repeated three times.

### 2.8 Immunohistochemistry and immunofluorescence (IF)

For immunohistochemistry (IHC), human colorectal cancer tissue microarray slides (Outdo Biotech, Shanghai, China, #HColA180Su08). Patient information provided by the TMA supplier. After the tissue sections were deparaffinized and rehydrated, antigen retrieval was carried out in citrate buffer (Beyotime) at 100°C for 0.5 hour. Endogenous peroxidase was blocked with 3% peroxide for 15 minutes and blocked in buffer (5% BSA +0.1% Triton X‐100) for 1 hour and incubated overnight in primary antibody. The primary antibodies used were anti‐DDX5 (Santa Cruz, #sc‐166167, 1:300), anti‐O‐GlcNAcylation (Abeam, #ab2739, 1:100), and detection of signals using a Vectastain ABC kit (Vector Labs, Burlingame, CA, USA). For IF, cells were fixed with 4% paraformaldehyde for 15 minutes, washed with PBS and blocking buffer (3% FBS +1% HISS +0.1% Triton X‐100), and then incubated overnight at 4°C in primary antibody. The primary antibodies used were anti‐DDX5 (Santa Cruz, #sc‐166167, 1:100), anti‐O‐GlcNAcylation (Abeam, #ab2739, 1:100). Fluorescent Alexa‐Fluor‐488 or ‐555‐conjugated secondary antibodies (Life technologies, Carlsbad, CA, USA) were used for detection.

### 2.9 Cell migration assay

Migration assays were performed in transwell plates (Corning, USA) according to the manufacturer's protocol. Briefly, cells were seeded at a density of 5 × 10^4^/well in a chamber at the top of a 24‐well plate in serum‐free medium. A total of 800 µl of medium containing 20% FBS was added to the bottom chamber. After 48 hours of incubation, the underside of the transwell membrane was fixed and stained with 0.05% crystal violet (Beyotime Biotechnology, Shanghai, China). The filter was observed using an inverted microscope (Olympus, Tokyo, Japan). The number of cells in each well was counted at 200x magnification in three random microscope fields per filter. Data were obtained from three separate experiments in duplicate.

### 2.10 mTOR agonist and inhibitor treatment

Rapamycin (Santa Cruz, sc‐3504) and MHY1485 (Sigma, SML0810) were dissolved in dimethyl sulfoxide (DMSO) as a stock solution at 1 and 5 mM, respectively. The cells were cultured in DMEM medium containing 10% FBS. When the number of cells reached 80%, cells were treated with rapamycin (50 nM) and MHY1485 (10 nM) for 12 hours.

### 2.11 Lentivirus production and infection

Stable infection SW480 that overexpresses OGT (SW480 LV‐OGT) or OGT knockdown by small hairpin RNA (SW480‐shOGT) by lentiviral infection using the GV341 vector (GeneChem Co., Ltd, Shanghai, China) Cell line. A stable infection SW480 cell line overexpressing DDX5 (SW480 LV‐DDX5) or DDX5 knockdown by small hairpin RNA (SW480‐shDDX5) was generated by lentiviral infection. An empty vector was used as a negative control (SW480 vector). A stably infected cell line overexpressing DDX5 (SW480‐shOGT +DDX5) was generated by lentiviral infection on the basis of SW480‐shOGT. The author's sequence is available upon request.

### 2.12 Tumorgenicity assay in nude mice

Nine male BALB/c nude mice (5 weeks old; Fourth Military Medical University, Xi'an, China) were randomly divided into three groups (SW480‐shOGT, SW480‐shOGT+DDX5 and vector group). All mice were maintained under conditions free of specific pathogens. This study was conducted in strict accordance with the recommendations of the National Institutes of Health Laboratory Animal Care and Use Guidelines. All procedures in the study involving animals were performed in accordance with the ethical standards of the Fourth Military Medical University. The cells were cultured and single cell suspensions were prepared from cells in the log phase. Cells (1 × 10^7^ cells) were injected subcutaneously into the right side of the back of nude mice to establish a subcutaneous xenograft tumour model. Mice were examined daily and bodyweight and tumour size were measured weekly. After 6 weeks, all mice were sacrificed under deep anaesthesia. The final volume and weight of each tumour was recorded. Tumour volume was calculated using the following formula: tumour volume (mm^3^) = [length (mm x width (mm)^2^] π/6.

### 2.13 Statistical analysis

Tests used to examine differences between groups included Student's *t* test, one‐way and two‐way ANOVA, and Chi‐squared test. *P* < 0.05 was considered statistically significant.

## 3 RESULTS

### 3.1 Correlation of DDX5 with O‐GlcNAcylation in vitro and in vivo

**Figure 1:**
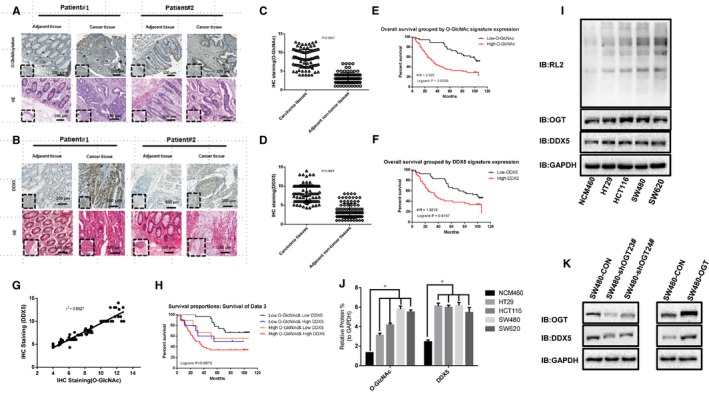
O‐GlcNAcylation and DDX5 are significantly up‐regulated in human colorectal cancer tissues and cell lines. A, Representative images of immunohistochemistry (IHC) staining of O‐GlcNAcylation and haematoxylin and eosin (HE) staining in 90 CRC tissues and 90 adjacent normal tissues. The high (upper) and low (lower) expression levels of O‐GlcNAcylation were evaluated by semi‐quantitative staining intensity (high score: 8‐12; low score 0‐6). B, Representative images of immunohistochemistry (IHC) staining of DDX5 and haematoxylin and eosin (HE) staining in 90 CRC tissues and 90 adjacent normal tissues. The high (upper) and low (lower) expression levels of O‐GlcNAcylation were evaluated by semi‐quantitative staining intensity (high score: 8‐12; low score 0‐6). C and D, Chi‐square analysis of O‐GlcNAcylation and DDX5 levels in 90 CRC tissues and 90 adjacent normal tissues. E and F, the Kaplan‐Meier curve depicts the overall survival of patients with CRC (n = 90). The curves were stratified based on O‐GlcNAcylation and DDX5 levels, respectively. The overall survival was defined as the interval between the date of surgery and death or the last follow‐up. G, The correlation coefficient (rs) between the scores of DDX5 and O‐GlcNAcylation was determined in the CRC tissue (*r* ^2^ = 0.8927). H, Kaplan‐Meier analysed concurrent O‐GlcNAcylation and DDX5 expression with recurrence and overall survival in CRC (n = 90) patients. I and J, O‐GlcNAcylation and DDX5 expression in established colonic epithelial cells (NCM460) and colon cancer cell lines were separately detected by WB. The levels of DDX5 and O‐GlcNAcylation were normalized to the level of GAPDH. The data are shown as the means + SD from three independent experiments (including WB). *P < 0.0001 indicates statistical significance. K, Knockdown of OGT can inhibit the level of DDX5, and overexpression of OGT increases the level of DDX5. The data are shown as the means + SD from three independent experiments (including WB)

To explore the correlation between DDX5 and O‐GlcNAcylation expression, tissue microarray (TMA) of human CRC tissue was used. The immunohistochemical (IHC) staining revealed that both DDX5 and O‐GlcNAcylation were significantly up‐regulated in CRC tissues compared to adjacent non‐tumour tissues (Figure 1A‐D). Higher O‐GlcNAcylation and DDX5 expression were not correlated with age, gender, or tumour location (*P* > 0.05, Table S2) but were significantly positively associated with lymph node metastasis, tumour size, and the American Joint Committee on Cancer (AJCC) status (*P* < 0.05, Table S2). Meanwhile, CRC patients with higher O‐GlcNAcylation expression showed shorter overall survival than patients with lower expression of O‐GlcNAcylation (Figure 1E, *P* < 0.01). Similarly, patients with higher DDX5 expression had shorter overall survival than those with lower DDX5 (Figure 1F, *P* < 0.05). Then, a statistically significant positive correlation between DDX5 expression and global O‐GlcNAcylation was analysed in 90 colorectal cancer samples using TMA analysis (Figure 1A, B, G). Kaplan‐Meier analysis showed that patients with higher DDX5/O‐GlcNAcylation co‐expression had the shortest overall survival. Meanwhile, patients with lower DDX5/O‐GlcNAcylation co‐expression had the longest overall survival (Figure 1H). Then, we further clarify whether there is also a correlation between DDX5 and O‐GlcNAcylation in colorectal cancer cell lines. We examined the expression of OGT, DDX5, and O‐GlcNAcylation in four colorectal cancer cell lines (HT29, HCT116, SW480, SW620) and the normal human intestinal epithelial cell line NCM460. As expect, the results of western blotting assay shown that the OGT, DDX5, and O‐GlcNAcylation levels were significantly increased in all cancer cell lines compared to that in the NCM460 cell line (Figure 1I and J). In addition, we found that knockdown of OGT resulted in a significant decrease in DDX5 levels (Figure 1K). Conversely, overexpression of OGT increases the level of DDX5, indicating that there may be a correlation between DDX5 and OGT (Figure 1K). In summary, our results indicate that DDX5 and O‐GlcNAcylation may be correlated in vivo and in vitro.

### 3.2 O‐GlcNAcylation promotes tumorigenesis via DDX5

**Figure 2:**
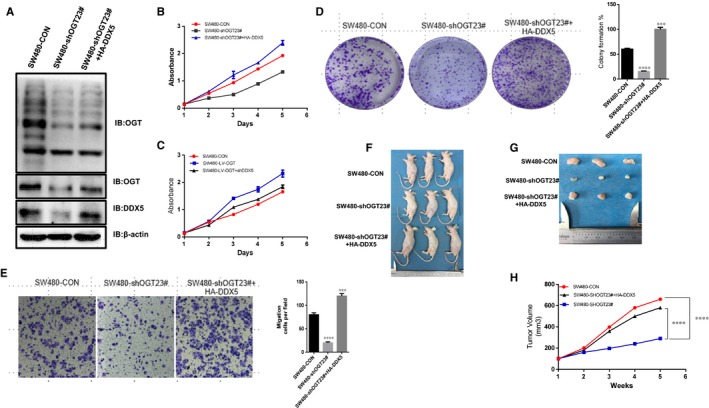
O‐GlcNAcylation promotes tumorigenesis via DDX5. A, B, C, D and E. Overexpression of DDX5 rescued a reduction in the transformed phenotype caused by OGT knockdown in vitro. A, Protein expression of OGT, O‐GlcNAcylation and DDX5 in SW480 cells detected by WB. B and C, Cell proliferation was measured using a CCK‐8 based assay. D, Cell proliferation was measured using an assay based on colony formation assay. E, Cell migrate ability was measured using a transwell based assay. F, G and H, Overexpression of DDX5 rescued xenograft tumour growth in vivo by OGT silencing. After three groups (SW480‐shOGT, SW480‐shOGT+DDX5, and vector group) cells were injected subcutaneously into nude mice, tumour volume was monitored for 6 wk; n = 3 per group. *P < 0.01, ***p < 0.001; ****p < 0.0001 indicates statistical significance. The data from B, C, D, E, and H were analysed by one‐way and two‐way ANOVA, respectively

It has been observed that elevated levels of O‐GlcNAcylation in CRC cells prompted us to investigate whether O‐GlcNAcylation has a tumour‐promoting function.^27^ We found that knockdown of OGT resulted in a significant decrease in DDX5 and O‐GlcNAcylation levels (Figure 2A) in SW480 cells, as well as the abilities of cell proliferation (Figure 2B), colony formation (Figure 2D), and migration (Figure 2E). Interestingly, we found that overexpression of DDX5 on the basis of OGT knockdown could partly rescue the abilities of cell proliferation (Figure 2B), colony formation (Figure 2D), and migration (Figure 2E). Meanwhile, knockdown of DDX5 can significantly block the effect of promoting cell proliferation which mediated by overexpression of OGT (Figure 2C). We also found that xenografts grew much more slowly in OGT knockdown group than control group, and overexpression of DDX5 also partly reversed the growth of xenografts (Figure 2F‐H). Collectively, these in vitro and in vivo results indicated that stimulation of cellular O‐GlcNAcylation can promote colorectal tumorigenesis in a DDX5‐dependent manner.

### 3.3 O‐GlcNAc modification increase DDX5 protein stability

**Figure 3:**
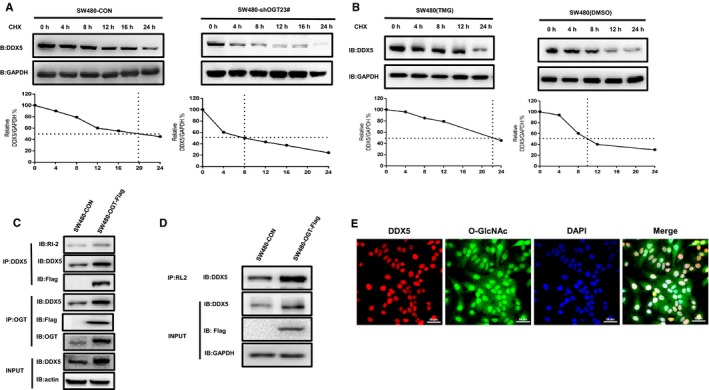
OGT interacts with DDX5 in colorectal cancer cell lines by O‐GlcNAcylation modification increase DDX5 protein stability. A, Protein synthesis was blocked by treatment with CHX (50 mg/mL) for the indicated time. The half‐life of endogenous DDX5 was used in SW480 cells infected with OGT‐specific shRNA and control cells at 24 h. B, Protein synthesis was blocked by treatment with CHX (50 mg/mL) for the indicated time. The half‐life of endogenous DDX5 in SW480 cells treated with DMSO or TMG (50 mg/mL) for 24 h. C, Total lysates from SW480 cells expressing OGT‐FLAG were IP‐precipitated with OGT Ab or DDX5 Ab and then Western blotted using the indicated antibodies (Abs). D, Total lysates from SW480 cells expressing OGT‐FLAG were IP‐prepared with O‐GlcNAcylation Ab (RL2) and then Western blotted using DDX5 Ab. E. Immunofluorescence colocalization in SW480 cells revealed that O‐GlcNAcylation can colocalize with DDX5. The data are expressed as the mean + SD from three separate experiments

We subsequently investigated how O‐GlcNAcylation regulates DDX5 expression levels. Firstly, we found that O‐GlcNAcylation did not affect DDX5 mRNA levels. (Figure S1A). Since O‐GlcNAcylation is reported to be an important factor affecting protein stability,^12^ we therefore investigated whether stimulation of O‐GlcNAcylation inhibits DDX5 degradation. To clarify whether down‐regulation of O‐GlcNAcylation alters the stability of DDX5, SW480 cells with or without OGT knockdown were treated with cycloheximide to inhibit protein synthesis, and then the remaining DDX5 was detected. As shown in Figure 3A, the protein level of DDX5 was reduced to 50% of its original amount in the cells of the control group at 18.5 hours after the cycloheximide treatment. However, in cells with OGT knockdown, DDX5 was reduced to 50% of its original amount at 7.5 hours after the cycloheximide treatment. Similar results showed that DDX5 protein levels were reduced to 50% at 22.5 hours after Thiamet‐G (TMG, O‐GlcNAcylationase inhibitors) treatment and at 12.5 hours after DMSO treatment. (Figure 2B). These results indicate that OGT regulates the stability of DDX5 mainly by modifying the DDX5 protein through O‐GlcNAcylation and increasing protein stability.

### 3.4 OGT interacts with DDX5 in CRC cell line

O‐GlcNAcylation is well known to be dynamically regulated by OGT and OGA.^22^ To elucidate the exact mechanism by which O‐GlcNAcylation promotes the development of colorectal cancer, co‐immunoprecipitation (Co‐IP) analysis was performed in SW480 OGT‐Flag overexpressing cells using OGT and O‐GlcNAcylation antibodies (Figure 3C, D). The results showed that DDX5 Co‐IP with OGT‐Flag in the interaction experiment (Figure 3C). Meanwhile, we Co‐immunoprecipitated DDX5 with anti‐O‐GlcNAcylation antibody in overexpressing OGT‐Flag cells and control cells, and then subjected to Western blotting. The results showed that endogenous DDX5 was also pulled down by O‐GlcNAcylation antibody (Figure 3D). In addition, immunofluorescence colocalization in SW480 cells revealed that O‐GlcNAcylation can colocalize with DDX5 (Figure 3E). Our results indicate that OGT can directly interact with DDX5 in colorectal cancer cell lines.

### 3.5 OGT‐DDX5 axis affects colorectal cancer proliferation and metastasis by regulating AKT/mTOR pathway

**Figure 4:**
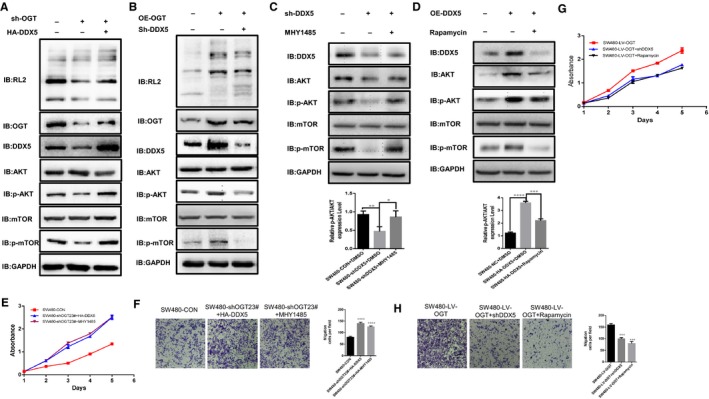
OGT‐DDX5 axis affects colon cancer proliferation and metastasis by regulating AKT/mTOR pathway. A, B, C, and D, The levels of O‐GlcNAcylation, OGT, DDX5, AKT, p‐AKT, mTOR, and p‐mTOR were detected by WB. A, WB detects the activation level of the AKT/mTOR signalling pathway after knockdown of OGT or overexpression of DDX5. B, WB detects the level of activation of the AKT/mTOR signalling pathway after overexpression of OGT or knockdown of DDX5. C, WB detects the activation level of the AKT/mTOR signalling pathway after knockdown DDX5 or adding MHY1485. D, WB detects the activation level of the AKT/mTOR signalling pathway after overexpressing DDX5 or adding Rapamycin. E, F, G, and H. The aberrantly activated AKT/mTOR signalling pathway rescued a reduction in the transformed phenotype caused by OGT knockdown in vitro. Representative images from three independent experiments are shown (A‐E). **P < 0.01 indicates statistical significance. The data from E‐H were analysed by one‐way and two‐way ANOVA, respectively

Previous studies have found that DDX5 can regulate the expression of AKT and phosphorylation in colorectal cancer, thus affecting the development of colorectal cancer.^7^ It has also been reported that DDX5 can regulate the phosphorylation activity of mTOR and S6K1 in gastric cancer, thereby affecting the progression of gastric cancer.^4^ We found that knockdown of OGT significantly inhibited AKT/mTOR signalling pathway activity (Figure 4A). Conversely, overexpression of OGT activates the AKT/mTOR signalling pathway (Figure 4B). Meanwhile, we found that knockdown DDX5 also significantly inhibited AKT/mTOR signal activation (Figure 4C). Similarly, overexpression of DDX5 activates the AKT/mTOR signalling pathway (Figure 4D). In summary, we propose to envision whether the OGT‐DDX5 axis regulates the proliferation and metastasis of colorectal cancer by activating the AKT/mTOR pathway. To demonstrate that the OGT‐DDX5 axis regulates colorectal cancer cell proliferation and metastasis by activating the AKT/mTOR pathway. We overexpressed DDX5 or added an mTOR agonist (MHY1485) in the OGT knockdown colorectal cancer cell line. The results indicated that the cell proliferation and metastasis ability of DDX5 overexpressing and mTOR agonists is significantly enhanced in OGT knockdown SW480 (Figure 4E, F). Similarly, we knocked down DDX5 on the basis of OGT overexpression or used the mTOR inhibitor (Rapamycin) significantly reduce the proliferation and metastasis of colorectal cancer cells (Figure 4G, H). Therefore, we found that OGT knockdown can significantly inhibit the expression of DDX5, thereby changing the phosphorylation activity of AKT and mTOR (Figure 4A). At the same time, we found that overexpression of DDX5 in OGT knockdown cells significantly reversed the inhibition of the AKT/mTOR pathway (Figure 4A). Similarly, we detected increased expression of DDX5 and activation of the AKT/mTOR signaling pathway in OGT overexpressing cell lines (Figure 4B). We also found that it significantly inhibited the activity of AKT/mTOR signalling pathway by knocking down DDX5 in the OGT overexpressing cell lines (Figure 4B).

To further verify that the OGT‐DDX5 axis regulates colorectal cancer progression through the AKT/mTOR pathway. We knocked down DDX5 in SW480 and found that it can significantly inhibit colorectal cancer proliferation (Figure S1B). However, the addition of the mTOR agonist (MHY1485) significantly reversed cell proliferation (Figure S1B). Similarly, we added a mTOR inhibitor (Rapamycin) to DDX5 overexpressing cell lines to reduce cell proliferation changes caused by DDX5 overexpression (Figure S1C). Notably, we found that AKT/mTOR activity was significantly reduced in DDX5 knockdown colorectal cancer cell lines, but the addition of mTOR agonist (MHY1485) significantly reversed the activation of AKT/mTOR signalling pathway. At the same time, the AKT/mTOR signalling pathway was significantly activated in DDX5 overexpressing cells and was significantly reversed after the addition of mTOR inhibitor (Rapamycin). In summary, we initially demonstrated that the regulation of colorectal cancer by the OGT‐DDX5 axis may be dependent on the AKT/mTOR signalling pathway.

## 4 DISCUSSION

Recent studies have shown that O‐GlcNAcylation can modify multiple groups of oncogenic factors, and that up‐regulation of O‐GlcNAcylation is involved in tumour malignancies.^22^, ^28^, ^29^, ^30^ For example, modifications of p53 and c‐Myc and O‐GlcNAcylation will increase their stability by blocking ubiquitin degradation.^16^, ^20^ In addition to stabilizing carcinogenic factors, O‐GlcNAcylation also regulates its transcriptional activity. For example, O‐GlcNAcylation of p65 at Thr322 and Thr352 is critical for p65 promoter binding.^31^, ^32^ O‐GlcNAcylation is an important post‐translational modification (PTM) of proteins that plays a cancer promoting role in several cancers, including colorectal cancer.^9^, ^18^, ^22^

Clinical studies have also found that higher levels of O‐GlcNAcylation are involved in the malignant clinicopathological features of various cancer patients.^12^, ^18^, ^33^, ^34^ In gastric carcinoma, poorly differentiated tumours (grades II and III) showed significantly higher expression of O‐GlcNAcylation than grade I tumours.^35^, ^36^ At the same time, we detected the expression of O‐GlcNAcylation and DDX5 in colorectal cancer tissues and matched normal controls. The results also showed that the levels of O‐GlcNAcylation and DDX5 were significantly increased in cancer tissues, and it was found to be positively correlated by the expression analysis of O‐GlcNAcylation and DDX5 in cancer tissues.

A recent study showed that DDX5 is significantly higher in colorectal cancer than normal tissues, and DDX5 is involved in phosphorylation of AKT and mTOR.^7^ It has also been reported that activated mTOR signalling leads to increased expression of OGT and O‐GlcNAcylation in colorectal cancer.^14^ Moreover, increased levels of O‐GlcNAcylation activate the mTOR signal, suggesting that there may be a feedback regulatory loop between the mTOR signalling pathway and O‐GlcNAcylation. Protein levels in cancer cells, although many studies have reported a correlation between O‐GlcNAcylation and the mTOR pathway, the mechanism by which O‐GlcNAcylation regulates mTOR activation in colorectal cancer remains unclear. In our study, we have shown that when OGT is silenced in CRC cell lines, the protein level of DDX5 is reduced. However, the mRNA level of DDX5 did not change significantly. Therefore, we speculate that OGT regulation of DDX5 may be post‐transcriptional regulation. We used YinOYang 1.2 Server (www.cbs.dtu.dk/services/YinOYang) 35 to predict the presence of O‐GlcNAcylation sites in DDX5, suggesting that DDX5 can be directly modified by O‐GlcNAcylation (Figure S1D). Therefore, we detected the stable binding products of OGT and DDX5 by co‐IP. And the level of O‐GlcNAcylation affects the stability of DDX5 protein.

There is growing evidence that it is significantly associated with tumorigenesis.^37^, ^38^ Recent studies have found that DDX5 stimulates the development of gastric cancer by activating mTOR signalling.^4^ Cross‐regulated colorectal tumorigenesis between mTOR signalling and O‐GlcNAcylation was recently reported.^14^ Interestingly, in the current study, there was no report that mTOR was modified by O‐GlcNAcylation. Therefore, we speculate that the OGT regulation of mTOR can be achieved in other ways. However, the way in which OGT regulates mTOR remains unclear and further research is needed.

Here, we prove that DDX5 can be O‐GlcNAcylation，and O‐GlcNAcylation of DDX5 enhances DDX5‐dependent colorectal tumorigenesis. Furthermore, we found that O‐GlcNAcylation stimulates DDX5 activation of the AKT/mTOR pathway. In summary, our results have demonstrated that higher O‐GlcNAcylation and DDX5 levels significantly contributed to tumour proliferation and metastasis and indicate a poor prognosis in patients with CRC. We also demonstrated that O‐GlcNAcylation can mechanically increase the activation of the AKT/mTOR signalling pathway by regulating the expression of DDX5, thereby increasing the proliferation and metastasis of colorectal tumour cell lines. Therefore, we proposed that inhibition of the OGT‐DDX5 axis may be a potential new therapeutic target for cancer therapy.

## CONFLICT OF INTEREST

The authors confirm that there are no conflict of interests.

## AUTHOR CONTRIBUTIONS

NW, MZJ, and YYH designed the experiments and wrote the manuscript. NW, MZJ, YYH, HML, CY, HL, JYC, QQH, and YZ performed the experiments. BX and XX were involved in experimental design, discussions, advice, and suggestions. All authors reviewed and approved the manuscript.
