# Supplementary figures and images for: O‐GlcNAcylation promotes colorectal cancer progression by regulating protein stability and potential catcinogenic function of DDX5
Source: J Cell Mol Med. 2018 Nov 28;23(2):1354–62. doi: 10.1111/jcmm.14038 (PMC6349181; doi:10.1111/jcmm.14038)

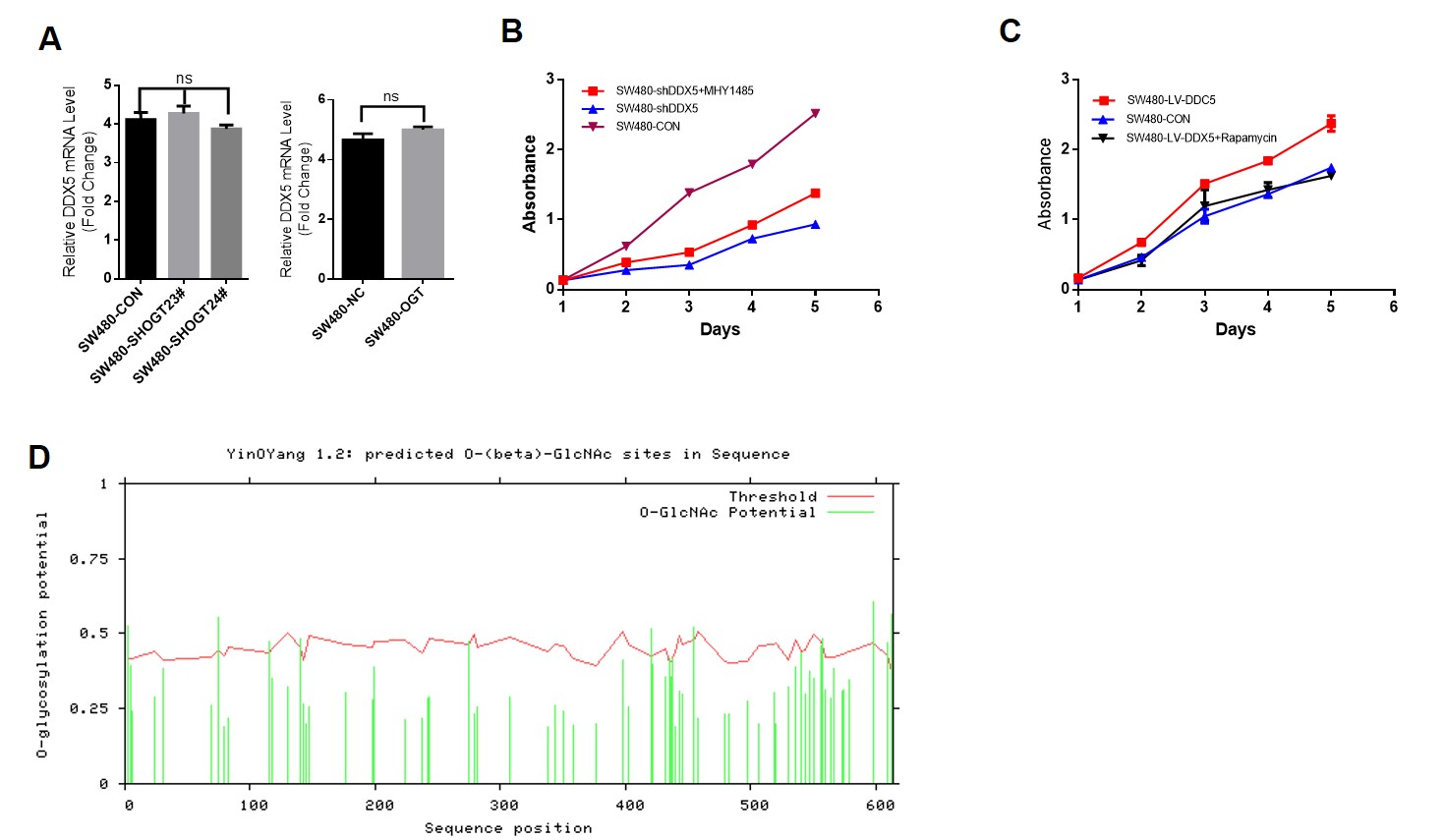

Supplement: Supplementary file 1 [file JCMM-23-1354-s001.jpg]

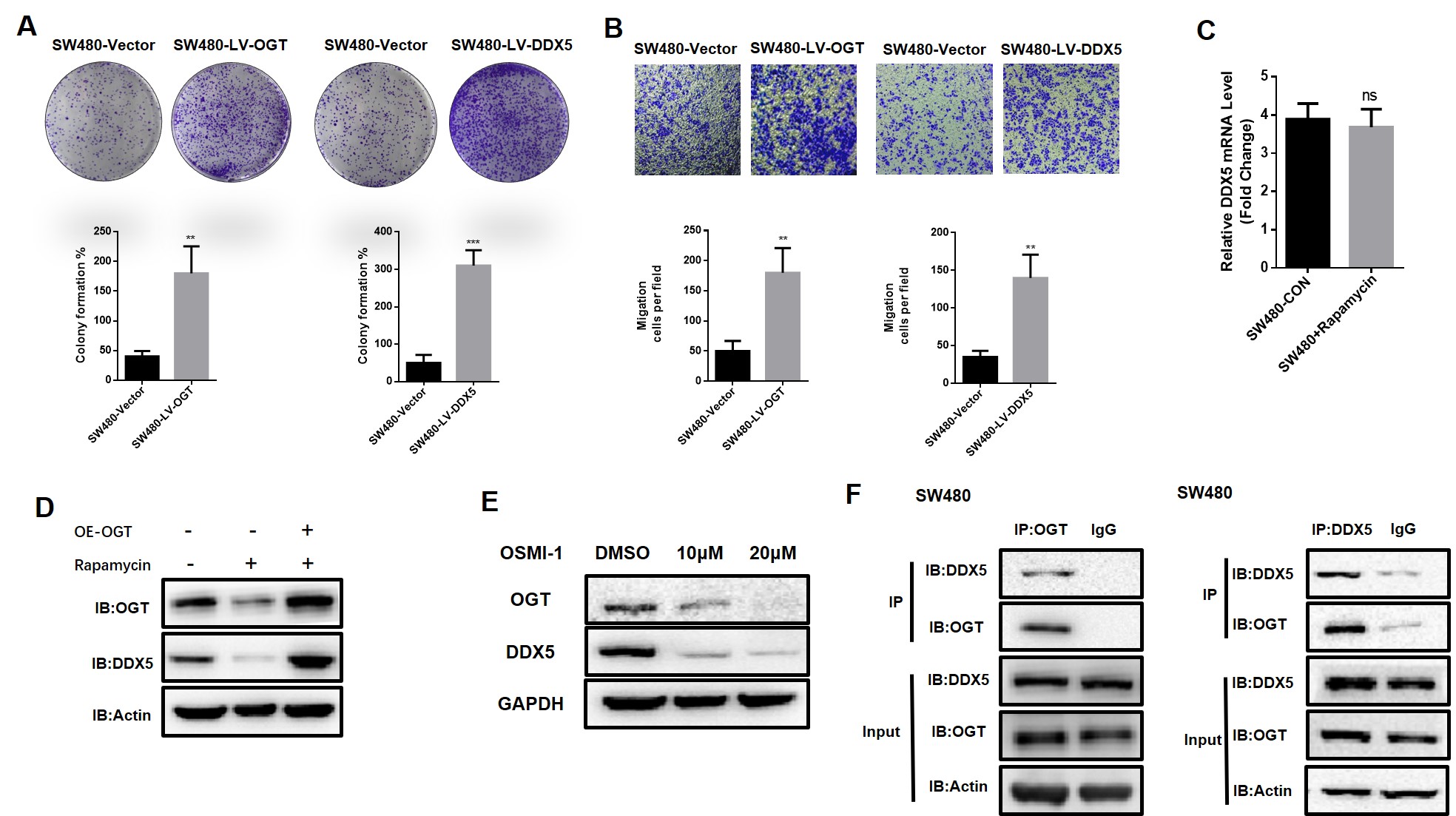

Supplement: Supplementary file 2 [file JCMM-23-1354-s002.jpg]
