# Supplementary material for: O‐GlcNAcylation promotes colorectal cancer progression by regulating protein stability and potential catcinogenic function of DDX5
Source: J Cell Mol Med. 2018 Nov 28;23(2):1354–62. doi: 10.1111/jcmm.14038 (PMC6349181; doi:10.1111/jcmm.14038)
Supplement: Supplementary file 3 [file JCMM-23-1354-s003.docx]

#### Supplemental Experimental Procedures:

#### Quantitative real-time polymerase chain reaction (qRT-PCR)

Total RNA from cells was extracted using an RNA isolation kit (Qiagen, Germany) according to the manufacturer's instructions. Subsequently, the RevertAid First Strand cDNA Synthesis Kit (TaKaRa, Japan) was used to reverse-transcribe the messenger RNA (mRNA) from the total mRNA; the specific primer (**Supplementary Table S1**) and the SYBR premix Ex Taq (TaKaRa, Japan) were used to expand by real-time qPCR. It was carried out with the following parameters: pre-denaturation at 95 ° C for 5 minutes, denaturation at 95 ° C for 10 seconds, annealing at 62 ° C for 20 seconds, and extension at 72 ° C for 30 seconds for 40 cycles. GAPDH was used as an internal control.

#### Cell viability assay

The cells were seeded in a 96-well plate (1 × 10^4^ cells/well), and cultured in a 37℃, 5% CO2 humidified incubator for 24 hours. Ten microliters of Cell Counting Kit-8 solution (Dojindo, Kumanoto, Japan) was added to each well and incubated for 2 hours at 37℃ in a 5% CO2 humidified incubator. Spectrometer Varioskan® Flash (Thermo Fisher, Waltham, USA) was used to measure absorbance at 450 nm. A proliferation curve is drawn in which time is taken as the abscissa and the average absorbance value in each group is taken as the ordinate. The experiment was performed in triplicate.

#### Supplemental Figure Legends：

**Supplementary Fig. S1.** **A.** O-GlcNAcylation does not regulate DDX5 mRNA levels by transcriptional level studies. **B and C.** Abnormally activated AKT/mTOR signaling pathways rescued a reduction in the transformed phenotype caused by DDX5 knockdown in vitro. **D.** The use of YinOYang 1.2 Server to predict the presence of the O-GlcNAcylation site in DDX5 suggests that DDX5 can be directly modified by O-GlcNAcylation.

**Supplementary Fig. S2**. **A.** Cell proliferation was measured using an assay based on colony formation assay. **B.** Cell migrate ability was measured using a transwell based assay. **C.** Rapamycin does not regulate DDX5 mRNA levels by transcriptional level studies. **D.** WB detects the activation level of the DDX5 after overexpressing OGT or adding Rapamycin. **E.** OSMI-1 does regulate DDX5 protein levels. **F.** Total lysates from SW480 cells were IP-precipitated with OGT Ab or DDX5 Ab and then Western blotted using the indicated antibodies (Abs). **p<0.01 indicates statistical significance. The data from A, B, C were analysed by one-way and two-way ANOVA, respectively.

**Supplementary Fig. S1**
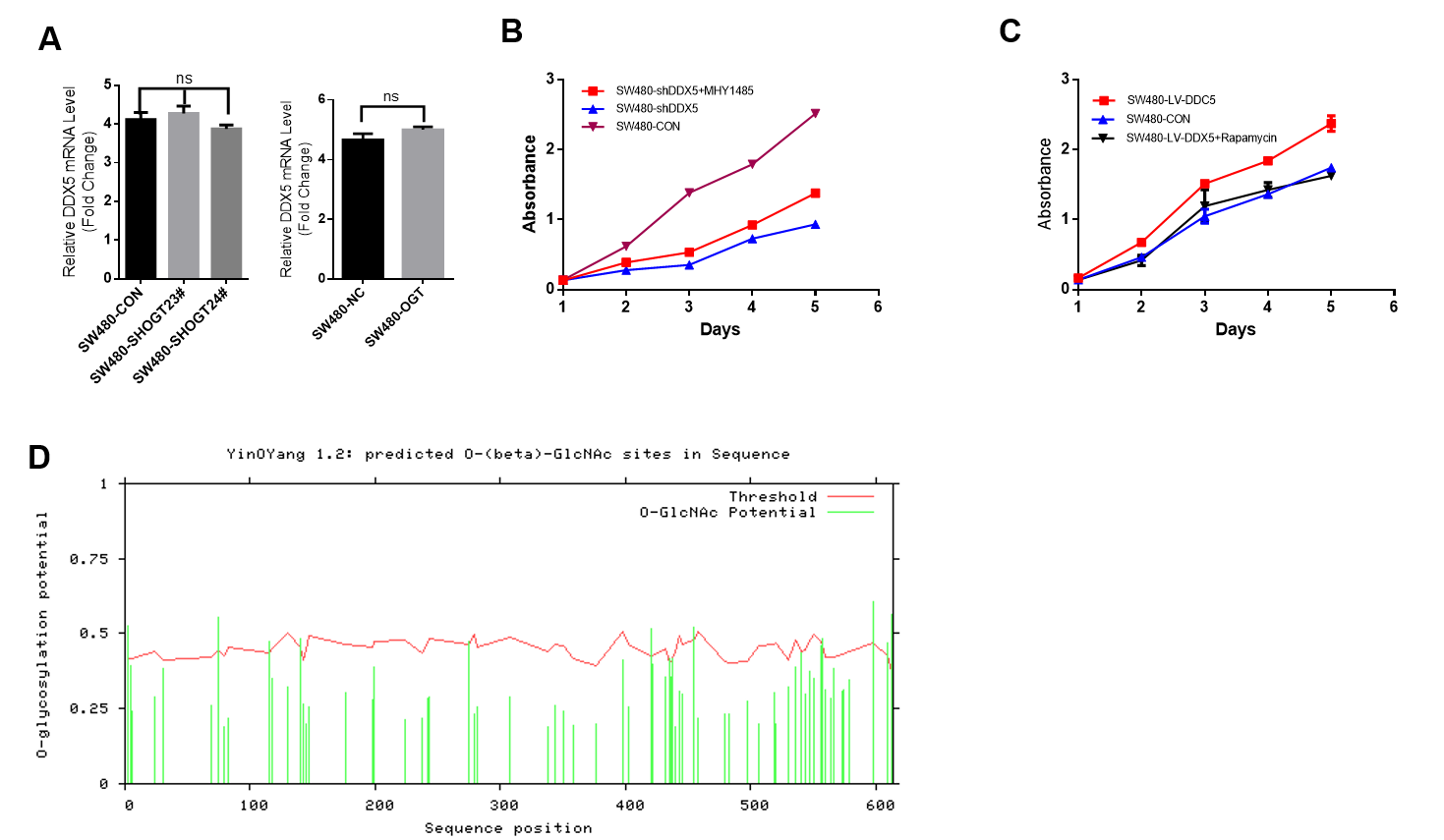


**Supplementary Fig. S2**


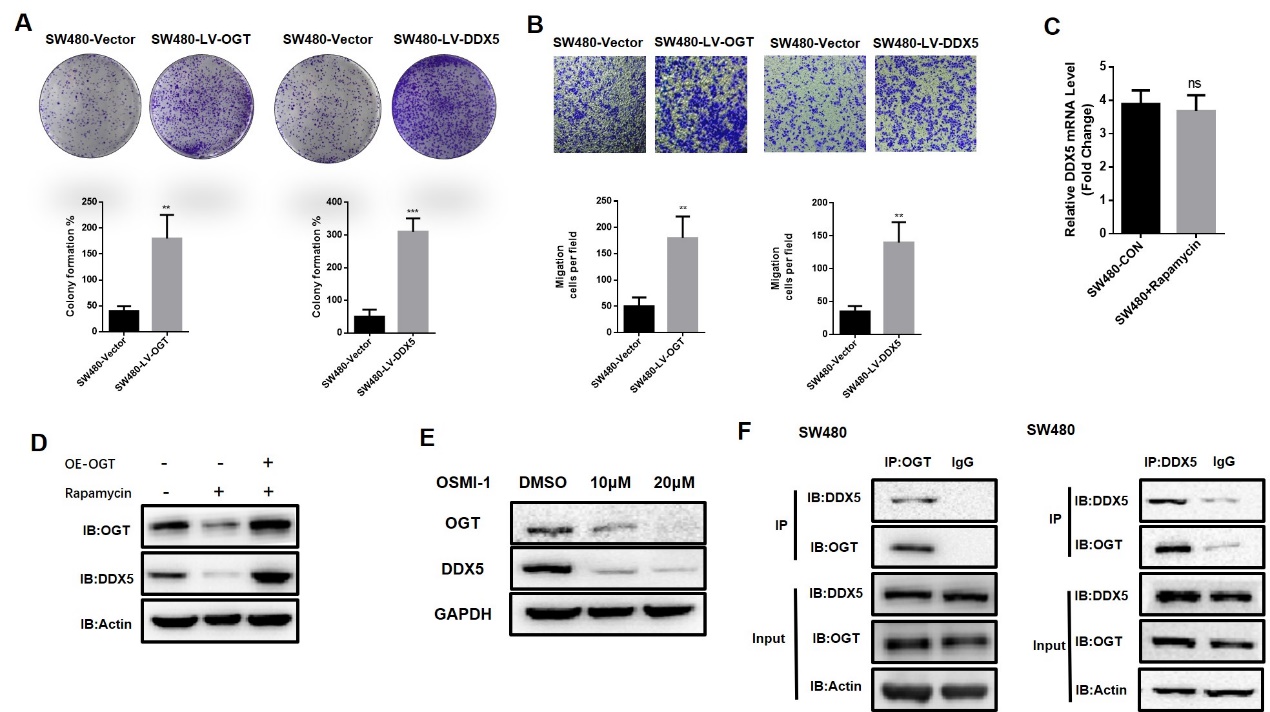


#### Supplementary Table S1

| **Primer** | **Sequence (5’-3’)** |
| --- | --- |
| **OGT-Forward** | **AGAAGGGCAGTGTTGCTGAAG** |
| **OGT-Reverse** | **TGATATTGGCTAGGTTATTCAGAGAGTCT** |
| **DDX5-Forward** | **GCCGGGACCGAGGGTTTGGT** |
| **DDX5-Reverse** | **CTTGTGCTGT GCGCCTAGCCA** |
| **GAPDH-Forward** | **GGAAGGTGAAGGTCGGAG TCA** |
| **GAPDH-Reverse** | **GTCATGATGGCAACAATATCCACT** |

**Table S1.** The list and sequences of primers used for qRT-PCR

#### Supplementary Table S2

**Table S2.** O-GlcNAc and DDX5 protein levels and clinicopathological features in 90 GC patients

| **Characteristics** | **Total** | **O-GlcNAc** | | **P value** | **DDX5** | | **P value** |
| --- | --- | --- | --- | --- | --- | --- | --- |
|  |  | **Low** | **High** |  | **Low** | **High** |  |
| **Gender** |  |  |  |  |  |  |  |
| **Female** | **43** | **18** | **25** | **0.890** | **16** | **27** | **0.605** |
| **Male** | **47** | **19** | **28** |  | **20** | **27** |  |
| **Age (years)** |  |  |  |  |  |  |  |
| **＞60** | **75** | **22** | **53** | **0.758** | **30** | **45** | **1.000** |
| **≤60** | **15** | **5** | **10** |  | **6** | **9** |  |
| **Tumor size(cm)** |  |  |  |  |  |  |  |
| **＜5** | **56** | **30** | **26** | **0.002** | **40** | **16** | **0.005** |
| **≥5** | **34** | **7** | **27** |  | **7** | **27** |  |
| **Invasion depth** |  |  |  |  |  |  |  |
| **Without Infiltration into Serous layer** | **15** | **11** | **4** | **0.03** | **12** | **3** | **0.005** |
| **Infiltration into Serous layer** | **75** | **30** | **45** |  | **25** | **50** |  |
| **Lymph node metastasis** |  |  |  |  |  |  |  |
| **Negative** | **21** | **16** | **5** | **0.001** | **18** | **7** | **0.001** |
| **Positive** | **69** | **27** | **42** |  | **25** | **44** |  |
| **Lymphatic and/or vascular invasion** |  |  |  |  |  |  |  |
| **Negative** | **66** | **36** | **30** | **0.03** | **35** | **31** | **0.02** |
| **Positive** | **24** | **7** | **17** |  | **4** | **20** |  |
| **AJCC stage** |  |  |  |  |  |  |  |
| **I\II** | **50** | **30** | **20** | **0.00005** | **31** | **19** | **0.00005** |
| **III\IV** | **40** | **7** | **33** |  | **6** | **35** |  |
| **O-GlcNAc** |  |  |  |  |  |  |  |
| **High** | **53** |  |  |  | **9** | **44** | **0.000095** |
| **Low** | **37** |  |  |  | **27** | **10** |  |
